# *Vital Signs:* Status of Human Immunodeficiency Virus Testing, Viral Suppression, and HIV Preexposure Prophylaxis — United States, 2013–2018

**DOI:** 10.15585/mmwr.mm6848e1

**Published:** 2019-12-06

**Authors:** Norma S. Harris, Anna Satcher Johnson, Ya-Lin A. Huang, Dayle Kern, Paul Fulton, Dawn K. Smith, Linda A. Valleroy, H. Irene Hall

**Affiliations:** ^1^Division of HIV/AIDS Prevention, National Center for HIV, Viral Hepatitis, STD, and TB Prevention, CDC; ^2^National Center for HIV, Viral Hepatitis, STD, and TB Prevention, CDC.

## Abstract

**Background:**

Approximately 38,000 new human immunodeficiency virus (HIV) infections occur in the United States each year; these infections can be prevented. A proposed national initiative, Ending the HIV Epidemic: A Plan for America, incorporates three strategies (diagnose, treat, and prevent HIV infection) and seeks to leverage testing, treatment, and preexposure prophylaxis (PrEP) to reduce new HIV infections in the United States by at least 90% by 2030. Targets to reach this goal include that at least 95% of persons with HIV receive a diagnosis, 95% of persons with diagnosed HIV infection have a suppressed viral load, and 50% of those at increased risk for acquiring HIV are prescribed PrEP. Using surveillance, pharmacy, and other data, CDC determined the current status of these three initiative strategies.

**Methods:**

CDC analyzed HIV surveillance data to estimate annual number of new HIV infections (2013–2017); estimate the percentage of infections that were diagnosed (2017); and determine the percentage of persons with diagnosed HIV infection with viral load suppression (2017). CDC analyzed surveillance, pharmacy, and other data to estimate PrEP coverage, reported as a percentage and calculated as the number of persons who were prescribed PrEP divided by the estimated number of persons with indications for PrEP.

**Results:**

The number of new HIV infections remained stable from 2013 (38,500) to 2017 (37,500) (p = 0.448). In 2017, an estimated 85.8% of infections were diagnosed. Among 854,206 persons with diagnosed HIV infection in 42 jurisdictions with complete reporting of laboratory data, 62.7% had a suppressed viral load. Among an estimated 1.2 million persons with indications for use of PrEP, 18.1% had been prescribed PrEP in 2018.

**Conclusion:**

Accelerated efforts to diagnose, treat, and prevent HIV infection are needed to achieve the U.S. goal of at least 90% reduction in the number of new HIV infections by 2030.

*On December 3, 2019, this report was posted online as an *MMWR *Early Release.*

## Introduction

Since 2013, progress in reducing the number of new human immunodeficiency virus (HIV) infections has stalled at approximately 38,000 new infections occurring each year (*1*). Infections are preventable. Persons who are aware that they have HIV infection and maintain a suppressed viral load (<200 copies of HIV RNA per mL) have effectively no risk of sexually transmitting the virus to HIV-negative partners (*2*). Nevertheless, 38% of new HIV infections are transmitted from persons with HIV infection who are unaware of their infection. Further, 43% of new HIV infections are transmitted from persons who have received a diagnosis but are not receiving HIV medical care, and 20% of new HIV infections are transmitted from persons receiving medical care for HIV, but who are not virally suppressed (*3*). Preexposure prophylaxis (PrEP), a daily oral pill that includes two HIV antiretroviral medications (tenofovir and emtricitabine), has been found to be highly effective in preventing acquisition of HIV infection (*4*). PrEP coverage has increased in recent years; however, coverage among persons at risk for exposure remains low (*5*). In February 2019, a new national initiative, Ending the HIV Epidemic: A Plan for America, was proposed. The plan calls for intensified efforts to diagnose, treat, and prevent HIV infections in the United States, with an overall goal of reducing new infections by at least 90% by 2030 (*6*). Use of PrEP is a major component of the prevention strategy and is indicated for men and women with sexual or injection drug use behaviors that increase their risk for acquiring HIV (*7*). To focus national and local prevention efforts on eliminating HIV, CDC analyzed surveillance, pharmacy, and other data to determine the status of these strategies (diagnose, treat, and prevent HIV infections) at the national and state levels.

## Methods

CDC analyzed data reported to the National HIV Surveillance System (NHSS) from the beginning of the epidemic in the early 1980s through June 2019 from 50 states and the District of Columbia (DC) for persons aged ≥13 years with diagnosed HIV infection. A CD4-depletion model* (*8*) was applied to NHSS data to estimate 1) the annual number of new HIV infections (2013–2017); 2) the total number of persons living with HIV (diagnosed and undiagnosed infection, or prevalence) at year-end 2017; and 3) the percentage of persons with HIV infection who had received a diagnosis.

NHSS data reported from 41 states and DC that had complete laboratory reporting of viral load test results were used to determine two viral suppression measures: viral suppression among persons with diagnosed HIV infection in the jurisdiction at year-end 2017 and viral suppression within 6 months of diagnosis among persons with HIV infection diagnosed during 2017. These 42 jurisdictions represent 89% of persons with diagnosed HIV infection in the United States.

CDC analyzed national pharmacy data from the IQVIA Real World Data–Longitudinal Prescriptions database to estimate the number of persons aged ≥16 years who were prescribed PrEP in 2017 and 2018. The annual number of PrEP prescriptions for persons aged ≥16 years was determined using an algorithm that included persons who had at least one tenofovir disoproxil fumarate and emtricitabine (TDF/FTC) prescription for >28 days and for whom TDF/FTC was not prescribed for HIV treatment, hepatitis B treatment, or HIV postexposure prophylaxis (*5*,*9*). NHSS, National Health and Nutrition Examination Survey, and U.S. Census data were used to estimate the number of persons aged ≥16 years with indications for PrEP (*10*). PrEP coverage, reported as a percentage, was calculated as the number of persons who were prescribed PrEP divided by the estimated number of persons who had indications for PrEP. To estimate PrEP coverage by race/ethnicity, the proportion among those with recorded race/ethnicity data was applied to those with missing race/ethnicity data. Analyses were conducted using SAS statistical software (version.9.4; SAS Institute).

## Results

The annual number of new HIV infections remained stable from 2013 (38,500) to 2017 (37,500) (p = 0.448). Among the estimated 1.2 million persons living with HIV infection in 2017, 85.8% (95% confidence interval [CI] = 84.3–87.5) had received a laboratory-confirmed diagnosis of HIV infection. The lowest percentages of diagnosed HIV infections were among persons aged 13–24 years (54.6%, 95% CI = 52.7–56.7), American Indians/Alaska Natives (79.5%, 95% CI = 58.7–100.0), and heterosexual males (82.0%, 95% CI = 76.5–88.3), compared with other age, racial/ethnic, or transmission risk groups. (Table 1). The percentage of diagnosed infections ranged from 79.7% in Nevada to 94.4% in New Jersey (Table 2).

**TABLE 1:** Percentage of diagnosed human immunodeficiency virus (HIV) infections, viral suppression among persons with diagnosed HIV infection, and prescription of preexposure prophylaxis (PrEP) for persons with indications, by demographic and transmission categories — United States, 2017 and 2018

Characteristic	2017		2018
	Diagnosed HIV infection,* % (95% CI)	Viral suppression,^†,§^ %	PrEP coverage,^¶,^**^,††^ %
**Sex**			
Male	84.9 (83.1–86.8)	63.3	20.8
Female	89.1 (86.1–92.3)	60.8	6.6
**Age group (yrs)**			
13–24	54.6 (52.7–56.7)	56.9	11.4
25–34	70.4 (69.4–71.4)	58.1	21.5
35–44	84.5 (83.6–85.4)	60.2	21.9
45–54	92.2 (91.5–92.9)	64.6	17.4
≥55	94.7 (93.9–95.5)	65.5	14.4
**Race/Ethnicity**			
American Indian/Alaska Native	79.5 (58.7–100.0)	62.0	—^§§^
Asian	83.7 (72.6–98.9)	68.3	—^§§^
Black/African American	85.5 (83.1–88.0)	57.4	5.9
Hispanic/Latino	83.0 (79.8–86.5)	62.3	10.9
Native Hawaiian/Other Pacific Islander	—*	65.0	—^§§^
White	88.6 (85.8–91.5)	69.3	42.1
Multiple races	86.7 (80.5–94.0)	69.9	—^§§^
**Transmission category**			
Male-to-male sexual contact	83.7 (81.7–85.8)	65.7	—^§§^
Injection drug use	93.8 (89.1–99.0)	—^¶¶^	—^§§^
Male	93.3 (87.0–100.0)	52.0	—^§§^
Female	94.4 (87.9–100.0)	58.4	—^§§^
Male-to-male sexual contact and injection drug use	92.0 (85.9–99.0)	63.1	—^§§^
Heterosexual contact	85.9 (83.0–89.0)	—^¶¶^	—^§§^
Male	82.0 (76.5–88.3)	57.6	—^§§^
Female	87.7 (84.4–91.2)	61.8	—^§§^
**Total**	85.8*** (84.3–87.5)	62.7***	**18.1**

**Abbreviation:** CI = confidence interval.

* Percentage of diagnosed infections calculated as the number of persons who received a diagnosis of HIV infection divided by the number of persons living with HIV (diagnosed and undiagnosed; n = 1,153,400). Dash in this column indicates estimate not available for some populations because of high relative standard errors.

^†^ Percentage viral suppression calculated as the number of persons with a viral load test result of <200 copies of HIV RNA per mL at last test divided by the number of persons living with diagnosed HIV infection (n = 854,206).

^§^ Includes data for 42 jurisdictions (41 states and District of Columbia) with complete laboratory reporting. These jurisdictions include Alabama, Alaska, California, Colorado, Connecticut, Delaware, District of Columbia, Florida, Georgia, Hawaii, Illinois, Indiana, Iowa, Louisiana, Maine, Maryland, Massachusetts, Michigan, Minnesota, Mississippi, Missouri, Montana, Nebraska, New Hampshire, New Mexico, New York, North Carolina, North Dakota, Ohio, Oklahoma, Oregon, Rhode Island, South Carolina, South Dakota, Tennessee, Texas, Utah, Virginia, Washington, West Virginia, Wisconsin, and Wyoming.

^¶^ PrEP coverage, calculated as the number of persons who were prescribed PrEP (n = 219,691 in 2018) divided by estimated number of persons with indications for PrEP (n = 1,211,777 in 2017).

** Total includes 1,605 persons prescribed PrEP with unknown jurisdiction and 143,168 persons prescribed PrEP with unknown/unavailable race/ethnicity. PrEP coverage for race/ethnicity was adjusted applying the distribution of records with known race/ethnicity to records with missing race/ethnicity.

^††^ Age group for PrEP coverage is 16–24 years.

^§§^ Dashes indicate data not available. IQVIA data source has incomplete race/ethnicity data and does not collect data on transmission risk category.

^¶¶^ Percentage viral suppression is presented for each sex within transmission category.

*** Total includes persons with HIV infection attributed to hemophilia, blood transfusion, perinatal exposure, or whose risk factor was not reported or not identified.

**TABLE 2:** Percentage of diagnosed human immunodeficiency virus (HIV) infections, viral suppression among persons with diagnosed HIV infection, and prescription of preexposure prophylaxis (PrEP) for persons with indications, by jurisdiction — United States, 2017 and 2018

Jurisdiction	2017		2018
	Diagnosed HIV infection,* % (95% CI)	Viral suppression,^†,§^ %	PrEP coverage,^¶,^** %
Alabama	83.9 (72.2–100.0)	57.3	13.2
Alaska	—*	78.7	8.3
Arizona	84.7 (74.1–98.8)	—^§^	13.1
Arkansas	82.2 (66.3–100.0)	—^§^	12.5
California	85.9 (81.6–90.5)	66.6	21.9
Colorado	85.8 (74.5–100.0)	58.6	13.3
Connecticut	88.6 (75.1–100.0)	66.8	21.3
Delaware	85.5 (64.9–100.0)	67.7	8.7
District of Columbia	88.6 (76.9–100.0)	56.0	36.5
Florida	87.0 (82.3–92.3)	63.0	11.1
Georgia	82.0 (76.0–89.1)	58.3	15.2
Hawaii	85.5 (63.1–100.0)	68.2	12.2
Idaho	96.6 (65.3–100.0)^††^	—^§^	10.0
Illinois	85.6 (77.9–94.9)	53.8	26.8
Indiana	83.8 (71.5–100.0)	61.3	10.1
Iowa	82.3 (61.6–100.0)	79.6	28.1
Kansas	84.0 (63.3–100.0)	—^§^	13.9
Kentucky	82.7 (68.3–100.0)	—^§^	9.2
Louisiana	81.2 (71.7–93.7)	64.7	22.8
Maine	85.9 (59.8–100.0)	78.3	11.9
Maryland	86.1 (78.1–95.9)	58.2	14.3
Massachusetts	89.5 (79.6–100.0)	70.9	33.4
Michigan	83.1 (72.2–97.9)	72.2	12.2
Minnesota	84.9 (71.8–100.0)	69.1	15.1
Mississippi	87.9 (73.8–100.0)	49.2	12.9
Missouri	85.2 (73.4–100.0)	66.2	14.2
Montana	—*	78.5	6.6
Nebraska	82.7 (59.8–100.0)	64.2	18.8
Nevada	79.7 (67.4–97.4)	—^§^	13.5
New Hampshire	85.5 (57.0–100.0)^††^	70.3	21.0
New Jersey	94.4 (85.6–100.0)	—^§^	16.8
New Mexico	81.2 (61.7–100.0)	68.5	12.0
New York	88.3 (84.0–93.0)	63.2	41.1
North Carolina	87.3 (79.0–97.5)	63.2	11.1
North Dakota	—*	77.7	14.8
Ohio	83.9 (74.8–95.5)	54.7	11.6
Oklahoma	82.9 (66.8–100.0)	59.0	7.6
Oregon	85.9 (71.4–100.0)	63.7	13.6
Pennsylvania	92.7 (84.6–100.0)	—^§^	22.9
Rhode Island	84.5 (62.2–100.0)	76.6	18.9
South Carolina	84.1 (73.9–97.5)	66.3	11.7
South Dakota	—*	47.0	11.3
Tennessee	84.9 (74.2–99.2)	57.6	11.4
Texas	81.1 (76.3–86.6)	61.3	14.3
Utah	81.9 (61.1–100.0)	62.5	21.9
Vermont	93.0 (59.0–100.0)^††^	—^§^	17.7
Virginia	86.9 (77.5–98.8)	55.2	9.5
Washington	88.3 (76.9–100.0)	78.6	25.0
West Virginia	86.9 (61.4–100.0)	58.9	9.7
Wisconsin	83.7 (68.3–100.0)	74.5	14.3
Wyoming	—*	76.8	5.0
Total	85.8 (84.3–87.5)	62.7	18.1

**Abbreviation:** CI = confidence interval.

* Percentage of diagnosed infections calculated as the number of persons who received a diagnosis of HIV infection divided by the number of persons living with HIV (diagnosed and undiagnosed). Dashes in this column indicate estimates not available for some jurisdictions because of high relative standard errors.

^†^ Percentage viral suppression calculated as the number of persons with a viral load test result of <200 copies of HIV RNA per mL at last test divided by the number of persons living with diagnosed HIV infection.

^§^ Includes data for 42 jurisdictions (41 states and District of Columbia) with complete laboratory reporting. These jurisdictions include Alabama, Alaska, California, Colorado, Connecticut, Delaware, District of Columbia, Florida, Georgia, Hawaii, Illinois, Indiana, Iowa, Louisiana, Maine, Maryland, Massachusetts, Michigan, Minnesota, Mississippi, Missouri, Montana, Nebraska, New Hampshire, New Mexico, New York, North Carolina, North Dakota, Ohio, Oklahoma, Oregon, Rhode Island, South Carolina, South Dakota, Tennessee, Texas, Utah, Virginia, Washington, West Virginia, Wisconsin, and Wyoming. Data were incomplete or not reported for nine jurisdictions, as indicated by dashes.

^¶^ PrEP coverage calculated as the number of persons who were prescribed PrEP (in 2018) divided by estimated number of persons with indications for PrEP (in 2017).

** Total includes 1,605 PrEP users with unknown jurisdiction.

^††^ Estimate does not meet the standard of reliability; use with caution.

In 2017, 62.7% of 854,206 persons with diagnosed HIV infections in 42 jurisdictions had a suppressed viral load (Table 1). The lowest percentages of persons with viral suppression were those aged 13–24 years (56.9%), blacks/African Americans (blacks) (57.4%), and males who inject drugs (52.0%), compared with other age, racial/ethnic, and transmission risk groups. The percentage of persons with a suppressed viral load ranged from 47.0% in South Dakota to 79.6% in Iowa (Table 2). The percentage of persons with a suppressed viral load within 6 months of diagnosis of HIV infection was 61.5 overall and <59% in 12 jurisdictions (Figure).

**FIGURE:**
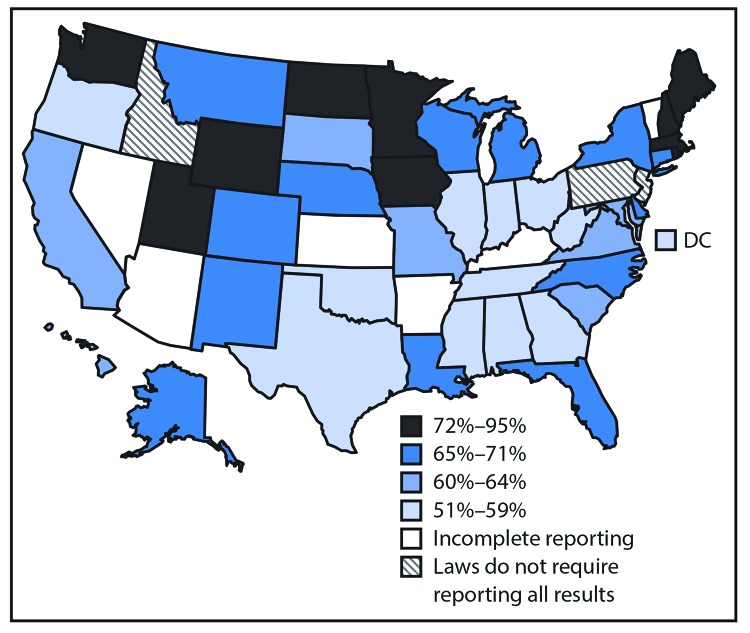
**Viral suppression** * ^,^ **^†,§^ within 6 months of diagnosis of human immunodeficiency virus (HIV) infection among persons aged ≥13 years — United States,^¶^ 2017** **Abbreviation:** DC = District of Columbia. * Percentage viral suppression within 6 months of HIV diagnosis, calculated as the number of persons with a viral load test result of <200 copies of HIV RNA per mL at last test divided by the number of persons with HIV diagnosed in 2017. Residence was based on residence at the time of diagnosis of HIV infection. ^†^ Total = 61.5%. ^§^ Data classified using quartiles. ^¶^ Analysis based on data reported from 41 states and DC; data for nine states were incomplete or not reported.

An estimated 1.2 million persons had indications for PrEP; 12.6% were prescribed PrEP in 2017 and 18.1% in 2018. In 2018, PrEP coverage was three times as high among males (20.8%) as among females (6.6%) (Table 1). Compared with other age groups, the lowest PrEP coverage rate was among persons aged 16–24 years (11.4%). Adjusting for missing race/ethnicity, PrEP coverage was 5.9% for blacks, 10.9% for Hispanics/Latinos, and 42.1% for whites. PrEP coverage ranged from 5.0% in Wyoming to 41.1% in New York (Table 2).

## Discussion

The annual number of new HIV infections has remained relatively stable since 2013. In 2017, the percentage of persons with HIV infection whose infection was diagnosed was 86%, a significant increase from 83% in 2010 (*1*). Overall, in 2017, 63% of persons with diagnosed HIV infection had a suppressed viral load, and in 2018, PrEP coverage was low at 18%. These findings confirm substantial gaps in diagnosing, treating, and preventing HIV infection and underscore the need for expanded efforts. The targets for the proposed initiative are at least 95% of persons with HIV infection having received a diagnosis, 95% of persons with diagnosed HIV infection having a suppressed viral load, and 50% of persons with indications for PrEP having been prescribed PrEP (*11*). New infections will occur unless substantial improvements are made in implementing these three strategies.

In this analysis, the lowest percentages of diagnosed HIV infection were among young persons (aged 13–34 years), American Indians/Alaska Natives, and heterosexual males. The low percentage of diagnosed HIV infection in these three populations might be explained by 1) lower testing rates among youths (*12*), 2) HIV-related stigma and lack of access to HIV-related services among American Indians/Alaska Natives (*13*), and 3) low patient and provider perceived risk for HIV acquisition among heterosexuals (*14*). The percentage of diagnosed HIV infections also varied geographically, possibly reflecting differences in access to and implementation of HIV testing and highlighting the need for developing tailored testing strategies (*15*). CDC recommends routine screening of all persons aged 13–64 years at least once in their lifetime (*16*), yet recent findings indicate that only 40% of persons aged ≥18 years in the United States have ever been tested for HIV (*15*). HIV testing guidelines also recommend at least annual testing for persons at high risk for acquiring HIV. Accelerating implementation of HIV testing strategies such as integrated and routinized HIV screening in health care settings, scaling up partner notification, social/sexual network screening, and mass distribution of HIV self-test kits (*15*) might facilitate early diagnosis.

The lowest percentages of viral suppression were found among young persons, blacks, and heterosexual males. Adherence to medication is critical to viral suppression. Factors associated with lower adherence or viral suppression include young age (*17*) and, for blacks, include health care coverage, homelessness, and incarceration (*18*). Expanded efforts must address these and other social and economic barriers to care. Developing or scaling up the implementation of evidence-based interventions is also important for improving adherence and viral suppression among youths and blacks. For example, one successful approach to improving viral suppression among blacks with HIV infection is an integrated care model that includes collaboration between community pharmacists and HIV medical care providers to develop individualized care plans that address HIV treatment challenges (*19*).

Since 2012, prompt treatment with antiretroviral therapy after diagnosis of HIV infection, regardless of stage of disease, has been recommended (*20*). Yet only 61.5% of persons with HIV infection diagnosed in 2017 had a suppressed viral load within 6 months of diagnosis. Low viral suppression rates within 6 months of HIV diagnosis (59%) occurred mainly in Southern states, which are already disproportionately affected by HIV (*1*). One study in patients with high rates of mental health illness, drug use, and housing instability illustrated success in reaching viral suppression within 1 year using multidisciplinary care and other support (*21*). To rapidly improve viral suppression for all populations, additional research is needed to identify interventions that will achieve viral suppression within 6 months of diagnosis, especially among populations facing severe health and socioeconomic challenges, including homelessness (*22*).

In 2019, the United States Preventive Services Task Force issued a Grade A recommendation^†^ that clinicians offer PrEP to persons at substantial risk for HIV acquisition (*4*). Overall, PrEP coverage was 9% in 2016 (*5*) and improved to 18% in 2018. Similar to earlier findings, PrEP coverage in this analysis was especially low in young persons (aged 16–24 years) compared with that in other age groups, and racial/ethnic and geographic disparities in PrEP prescription exist (*5*). In 2018, approximately 43% of HIV diagnoses were among blacks, and 26% were among Hispanics/Latinos (*23*). However, PrEP coverage among whites was seven times as high as that among blacks and four times as high as that among Hispanics/Latinos, suggesting that PrEP delivery to persons in racial/ethnic minority populations has not been equitable. Improving PrEP coverage will require targeted improvements in PrEP awareness, prescribing practices, and use in underreached demographic groups, especially among young persons, blacks, and Hispanics/Latinos at risk for acquiring HIV. CDC has developed a campaign, Prescribe HIV Prevention, which is designed to help clinicians provide PrEP to prevent acquisition of HIV (*24*).

The findings in this report are subject to at least three limitations. First, estimation of the number of new infections and percentage of undiagnosed infections relies on the assumption that persons received no treatment before their first CD4 test. The CD4 counts of persons with evidence of previous antiretroviral therapy use or viral suppression are excluded from the analysis, minimizing the impact of prior treatment on the HIV depletion model. Second, viral suppression measures in this analysis were based on data from 42 jurisdictions and are therefore not necessarily representative of data on all persons living with diagnosed HIV infection in the United States. Finally, although IQVIA recorded 92% of all prescriptions from retail pharmacies in the United States, prescriptions from closed health care systems (e.g., managed care organizations or military health plans) were not included. Therefore, these are minimum estimates of PrEP coverage. Different data sources were used in the numerator and denominator to calculate PrEP coverage. Although the result is expressed as a percentage, it is unknown whether all persons prescribed PrEP (numerator) are also contained in the denominator of the estimate of the number of persons with indications for PrEP. In addition, only 35% of persons with PrEP prescriptions identified in the IQVIA data had race/ethnicity information available. In calculating PrEP coverage, the racial/ethnic distribution of known records was applied to those for which data on race/ ethnicity were missing, which might not be valid. The extent to which the missing race/ethnicity is the same as that for those with reported race/ethnicity is unknown. Improvements in the completeness of race/ethnicity data in prescription databases are needed to fully describe disparities in PrEP coverage.

Accelerated efforts to diagnose, treat, and provide PrEP while addressing disparities, are urgently needed to reach the targets for the Ending the HIV Epidemic: A Plan for America initiative. These accelerated efforts, along with other prevention strategies such as quickly responding to increases in diagnoses of HIV infections, will be needed to meet the ambitious U.S. goal of at least a 90% reduction in the number of new HIV infections by 2030.

**Summary**

**What is already known about this topic?**

The approximately 38,000 new human immunodeficiency virus (HIV) infections that occur annually in the United States are preventable through testing, treatment, and preexposure prophylaxis (PrEP). A proposed initiative seeks to reduce new infections by at least 90% by 2030. The targets for the initiative are at least 95% for testing and treatment and 50% for PrEP.

**What is added by this report?**

In 2017, 85.8% of persons with HIV infection had received a diagnosis, and 62.7% of persons with diagnosed HIV infection had a suppressed viral load. In 2018, PrEP had been prescribed to 18.1% of persons with indications.

**What are the implications for public health practice?**

Accelerated efforts to diagnose, treat, and prevent HIV infection are urgently needed.

## Footnotes

* The first CD4 test result after HIV diagnosis and a CD4-depletion model indicating disease progression or duration after infection were used to estimate the number of new HIV infections and total prevalence (persons living with diagnosed or undiagnosed infection) among adults and adolescents in the United States.

† Grade A recommendation is a recommendation with high certainty that the net benefit of the intervention is substantial. https://www.uspreventiveservicestaskforce.org/Page/Name/grade-definitions.
